# Regression models for partially localized fMRI connectivity analyses

**DOI:** 10.3389/fnimg.2023.1178359

**Published:** 2023-11-13

**Authors:** Bonnie B. Smith, Yi Zhao, Martin A. Lindquist, Brian Caffo

**Affiliations:** ^1^Department of Biostatistics, Johns Hopkins Bloomberg School of Public Health, Baltimore, MD, United States; ^2^Department of Biostatistics and Health Data Science, Indiana University School of Medicine, Indianapolis, IN, United States

**Keywords:** fMRI, connectivity, connectomics, covariance regression, repeatability

## Abstract

**Background:**

Brain functional connectivity analysis of resting-state functional magnetic resonance imaging (fMRI) data is typically performed in a standardized template space assuming consistency of connections across subjects. Analysis methods can come in the form of one-edge-at-a-time analyses or dimension reduction/decomposition methods. Common to these approaches is an assumption that brain regions are functionally aligned across subjects; however, it is known that this functional alignment assumption is often violated.

**Methods:**

In this paper, we use subject-level regression models to explain intra-subject variability in connectivity. Covariates can include factors such as geographic distance between two pairs of brain regions, whether the two regions are symmetrically opposite (homotopic), and whether the two regions are members of the same functional network. Additionally, a covariate for each brain region can be included, to account for the possibility that some regions have consistently higher or lower connectivity. This style of analysis allows us to characterize the fraction of variation explained by each type of covariate. Additionally, comparisons across subjects can then be made using the fitted connectivity regression models, offering a more parsimonious alternative to edge-at-a-time approaches.

**Results:**

We apply our approach to Human Connectome Project data on 268 regions of interest (ROIs), grouped into eight functional networks. We find that a high proportion of variation is explained by region covariates and network membership covariates, while geographic distance and homotopy have high relative importance after adjusting for the number of predictors. We also find that the degree of data repeatability using our connectivity regression model—which uses only partial location information about pairs of ROI's—is comparably as high as the repeatability obtained using full location information.

**Discussion:**

While our analysis uses data that have been transformed into a common template-space, we also envision the method being useful in multi-atlas registration settings, where subject data remains in its own geometry and templates are warped instead. These results suggest the tantalizing possibility that fMRI connectivity analysis can be performed in subject-space, using less aggressive registration, such as simple affine transformations, multi-atlas subject-space registration, or perhaps even no registration whatsoever.

## 1. Introduction

Functional connectivity is defined as the undirected association between two or more brain regions (Friston, 2011). This is often assessed by computing the correlation coefficient over time in resting-state functional magnetic resonance imaging (fMRI) data between spatially different regions of the brain. In most functional connectivity studies, subjects' scans are first warped into a standard template, then analyzed using an assumption that the standardized data are functionally aligned across subjects. For example, edge-at-a-time approaches compare connectivity across subjects separately for each edge in the connectivity matrix, the matrix of correlations for each pair of brain regions. A shortcoming of such approaches is that the assumption of functional alignment is often violated: the presence of large inter-subject differences in functional localization is known to remain even after structural alignment to a standard template (Haxby et al., 2014). This problem can be partially addressed using spatial smoothing at a cost in spatial resolution, effect size, and power. Alternatively, it can be addressed using analytic approaches such as Hyperalignment (HA, Haxby et al., 2011, 2014, 2020). HA is a functional alignment technique that attempts to register individual brains based on functional properties rather than only on anatomical locations. It seeks to harness variation in functional connectivity to create a common functional space. Brain data from local regions are iteratively mapped into a common high-dimensional space using a Procrustes transformation, which preserves and aligns participants based on local representational geometry. This procedure has been shown to increase functional similarities across subjects while preserving subject-specific information (Haxby et al., 2011; Guntupalli et al., 2016; Feilong et al., 2018; Nastase et al., 2019). While hyperalignment and related procedures show great promise for allowing subjects to vary in their functional activation patterns, they are not shape-preserving. Hence, inference on location, size, and shape of activated areas is invalid. In addition, the solutions are not unique as it depends directly on the order in which individuals are entered into the algorithm.

Since edge-at-a-time approaches distinguish each individual edge in the connectivity matrix, they can be described as making a full use of the associated location information for each edge (that is, which pair of brain regions the edge corresponds to). In contrast, in recent work, Tang et al. (2023) treat all within-subject edges as exchangeable. Their approach ignores the location information for each edge and instead characterizes subjects by the distribution (or histogram) of their edges. This “edge distribution” has interesting properties, including being theoretically robust to registration. In addition, this approach is useful in settings such as in stroke, surgical, or degenerative disease settings where localization assumptions are both suspicious and difficult to employ because of registration challenges (Scheinost et al., 2012) (see Tward et al., 2020, for a discussion of registration in non-standard settings). However, since it does not use location information of the edges, this approach ignores the well-established core neuro-organizational principle of common localized functional specialization.

In this work, we propose an alternate approach that can be thought of as intermediate between the edge-at-a-time approach and the edge distribution approach. We use region-pair (or voxel-pair) information to explain within-subject connection strength using linear models, as is typically done in network analyses (Salter-Townshend and McCormick, 2017). For example, regression models could include covariates such as distance between the pair of regions and indicators of whether the two regions are members of the same functional network. We refer to our approach as being “partially localized” since the fitted connectivity values are based only on the partial location information contained in the covariates. This style of analysis allows us to compute the proportion of intra-subject variation explained by each type of covariate. Comparisons across subjects can then be made using the fitted connectivity regression models (see Section 4). Our regression approach offers a way of summarizing connectivity parsimoniously, using a much smaller number of parameters relative to the total number of edges. This can help alleviate the problem of multiple comparisons that arises with edge-at-a-time approaches. Further, since many different connectivity matrices will yield the same fitted model, our approach may be less sensitive to some violations of functional alignment than edge-at-a-time approaches, which use no summarization of the connections. In contrast to the edge distribution approach, connections are treated as exchangeable only within levels of the covariates. Additionally, connectivity regression models can be used either with data that has been transformed to a common template or in settings where each subject's data is left in its own geometry. For this latter point, we envision a use for this technique when using multi-atlas registration, where a single common template is not employed and instead template information is carried from a collection of labeled atlases to subjects (Rezende et al., 2019). Our proposed regression approach differs a great deal from functional HA approaches and related functional alignment techniques (Xu et al., 2012; Andreella et al., 2022; Wang et al., 2022) since: (i) explicit alignment is not a goal of the analysis; (ii) aggregate connectivity effects are considered under the assumption that they are exchangeable within levels of covariates; and (iii) in principle our approach can be implemented entirely in subject space using voxel-level data.

We illustrate our approach using resting-state fMRI data for healthy subjects from the Human Connectome Project (HCP, Van Essen et al., 2013). We summarize the fraction of variation explained by different groups of covariates used in our models, and we also investigate data repeatability (Finn et al., 2015; Airan et al., 2016; Bridgeford et al., 2021; Wang et al., 2021). Also known as test-retest reliability, data repeatability quantifies the consistency over time of multiple measurements made on the same subject. We compare data repeatability in the HCP data under our approach, the edge-at-a-time approach, and the edge distribution approach.

The paper is organized as follows: in Section 2, we describe the data, present our connectivity regression models, and describe how we assess data repeatability and proportion of variation explained by groups of covariates. In Section 3, we present results for the HCP data, and Section 4 concludes with a discussion.

## 2. Methods

### 2.1. Data from the Human Connectome Project

The dataset consists of resting-state fMRI data from 470 healthy subjects from the Human Connectome Project (HCP, Van Essen et al., 2013) 500 subject release. All data were acquired on a Siemens Skyra 3T scanner at Washington University in St. Louis. Subjects completed two fMRI sessions on consecutive days. Each session included two 15-min resting-state scans, one with a right-to-left and the other with a left-to-right phase encoding. In this work, we focus on the left-to-right phase encoding data, and hence our data consists of 1, 200 brain volumes (TR = 720 ms) for each day. An analysis of the right-to-left phase encoding data is also included in the Supplementary material. Further description of the data and processing pipelines applied can be found in Geuter et al. (2018). Briefly, scans were preprocessed using the HCP “fMRIVolume” pipeline (Glasser et al., 2013), which includes gradient unwarping, motion correction, distortion correction using FSL's topup tool, brain-boundary-based registration to structural T1-weighted scan, non-linear registration into MNI152 space, grand-mean intensity normalization, and spatial smoothing using a Gaussian kernel with a full-width half-maximum of 4 mm. This was followed by time series extraction using the Shen atlas of 268 regions of interest (Shen et al., 2013) via regional means. The Fisher's Z transform was taken of the synchronous temporal correlations across regions, resulting in 268 choose 2, or 35,778 transformed inter-regional correlations for each subject and session. Additionally, we also conducted a supplemental analysis in which data from the same subjects and sessions were extracted using the Glasser atlas of 360 regions of interest (Glasser et al., 2016), resulting in 64,620 inter-regional correlations for each subject and session (see the Supplementary material).

### 2.2. Subject-level connectivity regression models

We begin by defining notation. Let *Z*(*j, j*′) denote the Fisher's Z-transformed empirical correlation over time between two regions *j* and *j*′. Let *A* be the connectivity matrix, that is, the symmetric *R*×*R* matrix whose entries are the correlations *Z*(*j, j*′), where *R* is the number of region or seed locations (e.g., *R* = 268 using the Shen atlas). We also refer to each correlation *Z*(*j, j*′) as an edge of the connectivity matrix *A*. Let **Z** be the ordered vector of the *R* choose two correlations *Z*(*j, j*′) with *j* < *j*′, obtained as the vectorized strictly upper triangular elements of *A*. Additionally, let *Ƶ* = {*Z*(*j, j*′):*j* < *j*′} be the collection of the *R* choose two correlations viewed as an (unordered) set. All of these refer to subject- and session-specific measurements; when comparing multiple subjects and sessions, we will write $Z_{i,t}\left(j,j^{\prime }\right)$, *A*_*i, t*_, **Z**_*i, t*_, and *Ƶ*_*i, t*_ to denote measurements for subject *i*, session *t*.

Edge-at-a-time approaches compare each edge *Z*(*j, j*′) across subjects, while the edge distribution approach is based on *Ƶ*, the collection of edges not distinguished according to their location information. Here we propose using subject-level regression models to explain variation in connectivity. Separately for each subject, the vector of Fisher-transformed correlations **Z** is modeled in terms of characteristics of the pairs of regions. For the HCP data, we use the following as our main model; each term is explained below along with its rationale.


(1) $$\begin{array}{l} Z(j,j^{\prime })=\beta_{0}+s_{0}(\text{GeogDist}(j,j^{\prime });{\text{Hem}}_{0})\\ \;+s_{1}(\text{GeogDist}(j,j^{\prime });{\text{Hem}}_{1})\\ \;+s_{2}(\text{HomotopDist}(j,j^{\prime });{\text{Hem}}_{1})+\sum_{r=1}^{268}\beta_{r}\;{\text{Region}}_{r}(j,j^{\prime })\\ \;+\sum_{k=1}^{8}\sum_{k^{\prime }\geq k}\gamma_{k,k^{\prime }}\;{\text{Network}}_{k,k^{\prime }}(j,j^{\prime })+\epsilon (j,j^{\prime }). \end{array}$$

Here GeogDist(*j, j*′) is the geographic (i.e., Euclidean) distance between the centers of two regions (ROI's) *j* and *j*′. Geographic correlations can arise from biologically irrelevant reasons such as processing (smoothing), as well as biological reasons such as functional specialization. The model uses smooth terms of geographic distance, which were fit using generalized additive models (GAMs Wood, 2011; Hastie, 2017) with thin plate regression splines. Separate smooth terms for geographic distance are used for region-pairs that are in the same hemisphere and region-pairs that are in opposite hemispheres, denoted by $s_{0}\left(\text{GeogDist}\left(j,j^{\prime }\right);{\text{Hem}}_{0}\right)$ and $s_{1}\left(\text{GeogDist}\left(j,j^{\prime }\right);{\text{Hem}}_{1}\right)$, respectively.

HomotopDist(*j, j*′) is a measure of approximate symmetry (homotopy) between regions *j* and *j*′—specifically, the distance between one region-center, and the reflection across the mid-sagittal plane of the other. The rationale for including this term is that homotopic correlations are among the most reproducible resting-state findings (Zhao H. et al., 2022). The term $s_{2}\left(\text{HomotopDist}\left(j,j^{\prime }\right);{\text{Hem}}_{1}\right)$ denotes a smooth function of homotopic distance, among region-pairs that are in opposite hemispheres. (No term for same-hemisphere pairs is used since such pairs would not be symmetrically opposite.)

The term ${\text{Region}}_{r}\left(j,j^{\prime }\right)$ is an indicator that region *r* is involved in the pair—that is, that one of *j* or *j*′ is region *r*. The rationale for these region terms is the possibility that specific ROI's may exhibit consistently higher or lower connectivity. By including an indicator term for each ROI in our model, we also ensure that the average of the fitted values for pairs involving a given region is equal to the average connectivity on those pairs.

The final set of predictors uses network information based on the results of Finn et al. (2015), who group the 268 regions in the Shen parcellation into eight functional networks. For *k* = *k*′, the term ${\text{Network}}_{k,k}\left(j,j^{\prime }\right)$ is an indicator that regions *j* and *j*′ are both in network *k* (an intra-network indicator). For *k* < *k*′, ${\text{Network}}_{k,k^{\prime }}\left(j,j^{\prime }\right)$ is an indicator that one of region *j* or *j*′ is in network *k* and the other is in network *k*′ (an inter-network indicator). Thus, our model includes eight same-network membership terms and 28 different-network terms.

**Figure 1:**
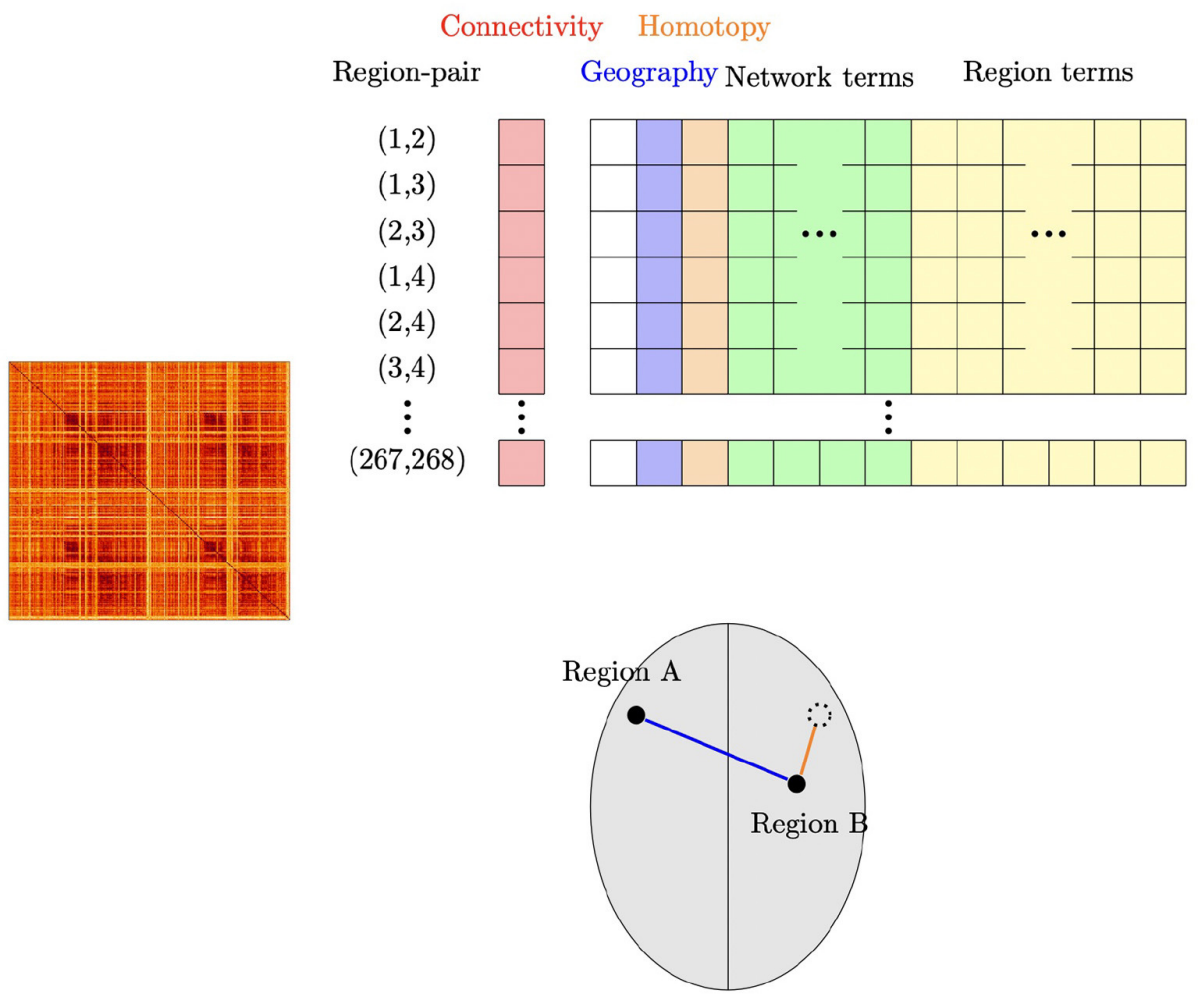
Schematic illustration of our subject-level connectivity regression model. The outcome vector (“Connectivity”) contains the Fisher Z-transformed correlation for each pair of ROI's, obtained from the upper triangular part of the subject's connectivity matrix shown at the left. For our main model using the Shen parcellation with 268 ROI's and eight functional networks, the design matrix contains an intercept; the geographic distance between the pair of ROI's; homotopic distance between the pair of ROI's; 36 network membership terms, indicating which network or pair of networks the two ROI's are members of; and 268 region terms, indicating which ROI's are in the pair. The lower part of the figure shows an example of two regions A and B; suppose that Region A is in Network 3 and Region B is in Network 2. The row corresponding to this pair of regions would have the geographic distance that is shown in blue; the homotopic distance (how far the regions are from being symmetrically opposite each other) shown in orange; an indicator that Region A is involved in the pair; an indicator that Region B is involved in the pair; and an indicator that this pair of regions are in Networks 2 and 3.

In summary, region-pair correlations are modeled using an intercept term and four different types of region-pair characteristics: (i) geographic distance between region-centers, (ii) homotopic distance (approximate symmetry) between region-centers, (iii) indicators of involvement of a given ROI, and (iv) membership in a given functional network or pair of functional networks. For brevity we refer to these four types of predictors as geographic distance, homotopic distance, region terms, and network membership terms. Including all 268 region terms and all 36 network membership terms introduced rank deficiency, so (as usual) terms were dropped to obtain a full-rank design matrix. Figure 1 shows a schematic illustration for this model. We also consider a number of other related models (see Section 2.5).

It is also worth emphasizing that this approach is a form of matrix regression (Zhao et al., 2021; Zhao Y. et al., 2022). Specifically, the fitted model minimizes the loss function $\underset{j<j^{\prime }}{\sum }{\left\{Z\left(j,j^{\prime }\right)-X{\left(j,j^{\prime }\right)}^{T}\beta \right\}}^{2}$ over β, where *X*(*j, j*′) is a vector containing an intercept term and characteristics of the region-pair (*j, j*′), and β is the vector of regression coefficients. The regression estimates for β are the same as those obtained by minimizing the norm $||Z-W_{1}\beta_{1}-\ldots -W_{p}\beta_{p}|{|}_{F}^{2},$ where *Z* is the matrix obtained from the connectivity matrix *A* by setting the diagonal entries to 0, the *W*_*k*_ are matrices with element (*j, j*′) equal to element *k* of *X*(*j, j*′), β_*k*_ is element *k* of β, and ||·||_*F*_ is the Frobenius norm (that is, the square root of the sum of the squares of the matrix elements).

We denote the fitted value for *Z*(*j, j*′) by Ẑ(*j, j*′) and the vector of fitted values by $\hat{\text{Z}}$.

### 2.3. Proportion of variation explained by groups of predictors

A substantial benefit of the proposed model is that it allows us to investigate the relative importance of each of the characteristics described above. For linear models, Grömping (2006) has presented a number of relative importance metrics, which are implemented in the relaimpo R package. The importance of a given predictor can be assessed using the amount by which the coefficient of determination *R*^2^ increases when that predictor is added to the model. However, this amount will typically depend on whether other predictors have already been included in the model. To account for this, the recommended lmg metric of Grömping (2006) averages the increase in *R*^2^ over all possible orderings of the predictors. In this way, a given predictor's relative importance weighs both the direct impact of that predictor on *R*^2^ when it is the only predictor in the model and the indirect impact when it is added after one or more of the other predictors. Here we adapt this for our setting, where we focus on the relative importance of groups of predictors in a generalized additive model (with Gaussian family and linear link). Specifically, we consider all of the network membership terms as one group, all of the region terms as a second group, and geographic distance and homotopic distance as the third and fourth groups of predictors, respectively. We then use the increase in *R*^2^ when a given group of predictors is added, and average over possible orderings of the four groups. For each subject-specific model, this partitions the total proportion of variation explained by the model into the proportion of variation explained by each of these four groups of predictors.

The proportion of variation explained by a group of predictors will be impacted by the number of predictors in the group, as well as the relative importance of each predictor. Therefore, for each group of predictors, we also consider *per-predictor relative importance*, which we define as the proportion of variation explained by the group of predictors divided by the number of predictors in the group. We use this metric rather than considering relative importance for each predictor individually (as is done in the relaimpo package) since this would involve fitting models on all possible orderings of the individual predictors, which would be prohibitively computationally expensive when using hundreds of predictors.

### 2.4. Data repeatability

We consider the degree of repeatability obtained by summarizing subjects using our connectivity-regression framework. Specifically, we investigate the degree of repeatability obtained if we use each subject's vector of fitted connectivity values $\hat{\text{Z}}$, compared with repeatability using the vector of Fisher-transformed correlations **Z** under the edge-at-a-time approach, or the set of correlations *Ƶ* under the edge distribution approach. We do this using the notion of *discriminability* (Bridgeford et al., 2021). Population discriminability is the probability that two measurements for the same subject will be more similar than two measurements from different subjects, under a given distance metric. To elaborate, let *M*_*i*_*t*__1__ and *M*_*i*_*t*__2__ be measurements for the same subject at two different sessions and let $M_{i^{\prime }t^{\prime }}$ be a measurement for a different subject at a possibly different session. For a given distance *d*(·, ·) compatible with the measurements, discriminability is the probability $\delta =Pd\left(M_{it_{1}},M_{it_{2}}\right)<d\left(M_{it_{1}},M_{i^{\prime }t^{\prime }}\right)$, which is well-defined assuming independent subjects and that this probability does not depend on the specific subjects and sessions being considered (Wang et al., 2020). Given *n* independent subjects each measured at *T* sessions, a consistent and unbiased estimator is given by the sample discriminability $\hat{\delta }$ (Wang et al., 2020; Bridgeford et al., 2021):


(2) $$\begin{array}{l} \hat{\delta }\;=\;\frac{1}{n(n-1)T^{2}(T-1)}\;\times \\ \sum_{i=1}^{n}\sum_{i^{\prime }\neq i}\sum_{t_{1}=1}^{T}\sum_{t_{2}\neq t_{1}}\sum_{t^{\prime }=1}^{T}I\left\{d(M_{it_{1}},M_{it_{2}})<d(M_{it_{1}},M_{i^{\prime }t^{\prime }})\right\}, \end{array}$$

where *I*{·} is the indicator function.

We note that, when comparing discriminability across different types of measurements, different distances may be needed for the different measurements. The different choices of distances will impact discriminability of the different measurements. We see this dependence as a positive feature, correctly reflecting the degree of repeatability we have for each measurement, since the question of interest is how well we are able to distinguish within-subject vs. between-subject measurements by using a distance appropriate for the type of data at hand. For measurements that are ordered vectors, as we have in the edge-at-a-time approach and our connectivity regression approach, we use Euclidean distance. For the edge distribution approach, each measurement is an (unordered) set, so Euclidean distance cannot be used. Instead, we use the 2-Wasserstein distance (see Galichon, 2018) between *Ƶ*_*i, t*_ and ${Ƶ}_{i^{\prime },t^{\prime }}$, which is simply the Euclidean distance between the vectors of order statistics for *Ƶ*_*i, t*_ and for ${Ƶ}_{i^{\prime },t^{\prime }}$.

An alternative way of measuring discriminability under our approach would take the measurement for each subject to be the vector of regression coefficients, rather than the vector of fitted values. Euclidean distance using the fitted values is a Mahalanobis distance of the coefficients around the variance/covariance matrix of the regressors, while Euclidean distance using the regression coefficients is a Mahalanobis distance around an identity matrix. Using the regression coefficients as the measurement could be preferable in some ways, since it moves away from comparing the fitted values separately for each edge, as in the edge-at-a-time approach. However, comparing the fitted values as we have done here has the benefit that the fitted values are fixed in dimension across different models and independent of the units used.

### 2.5. Approaches and models compared

In Section 2.2, we presented our main connectivity regression model (Equation 1), in which region-pair correlations are modeled using an intercept term and four different types of predictors: geographic distance, homotopic distance, region terms, and network membership terms. We also consider models that use subsets of these predictors. All models contain an intercept term and the geography and homotopy terms of (1). Our smallest model (which we denote as “Reference model”) uses only these predictors. Two other models, denoted as “Reference model + networks” and “Reference model + regions,” additionally include the network membership terms or the region terms. The main model including all of these predictors we also denote as “Reference model + networks + regions.” We analyze the HCP data with the Shen parcellation using each of these connectivity regression models, and investigate the proportion of variation explained by each group of predictors used in each model. We then compare discriminability under the edge-at-a-time approach, under each of these connectivity regression models, and under the edge distribution approach. A supplementary analysis using different parcellations is also described in the Supplementary material.

## 3. Results

**Figure 2:**
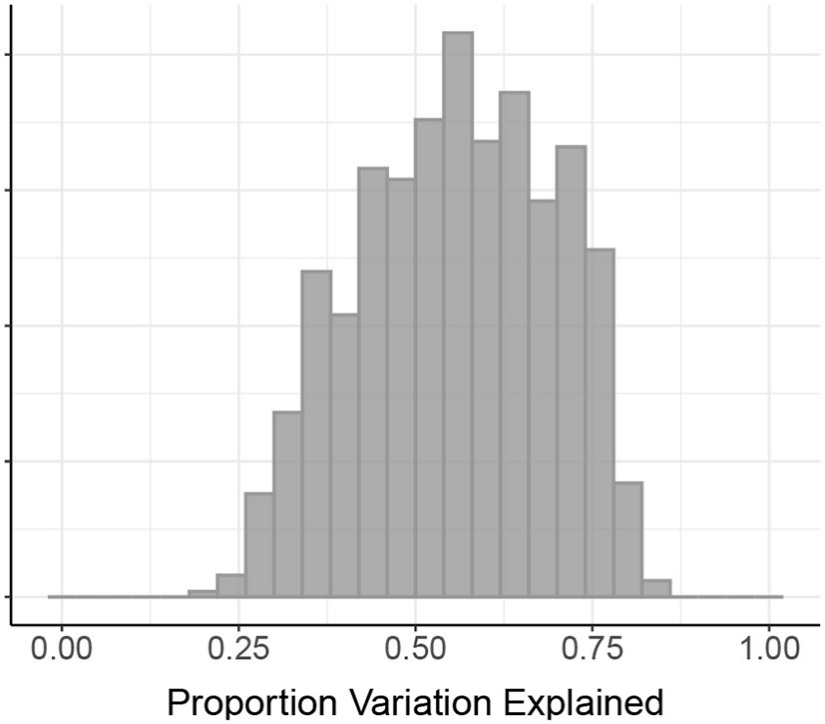
Proportion of variation in connectivity in the HCP data explained by our main connectivity regression model. The proportion of variation explained is shown for each of the 470 subjects and two sessions in the HCP data. The median across all subjects and sessions is 0.56.

**Figure 3:**
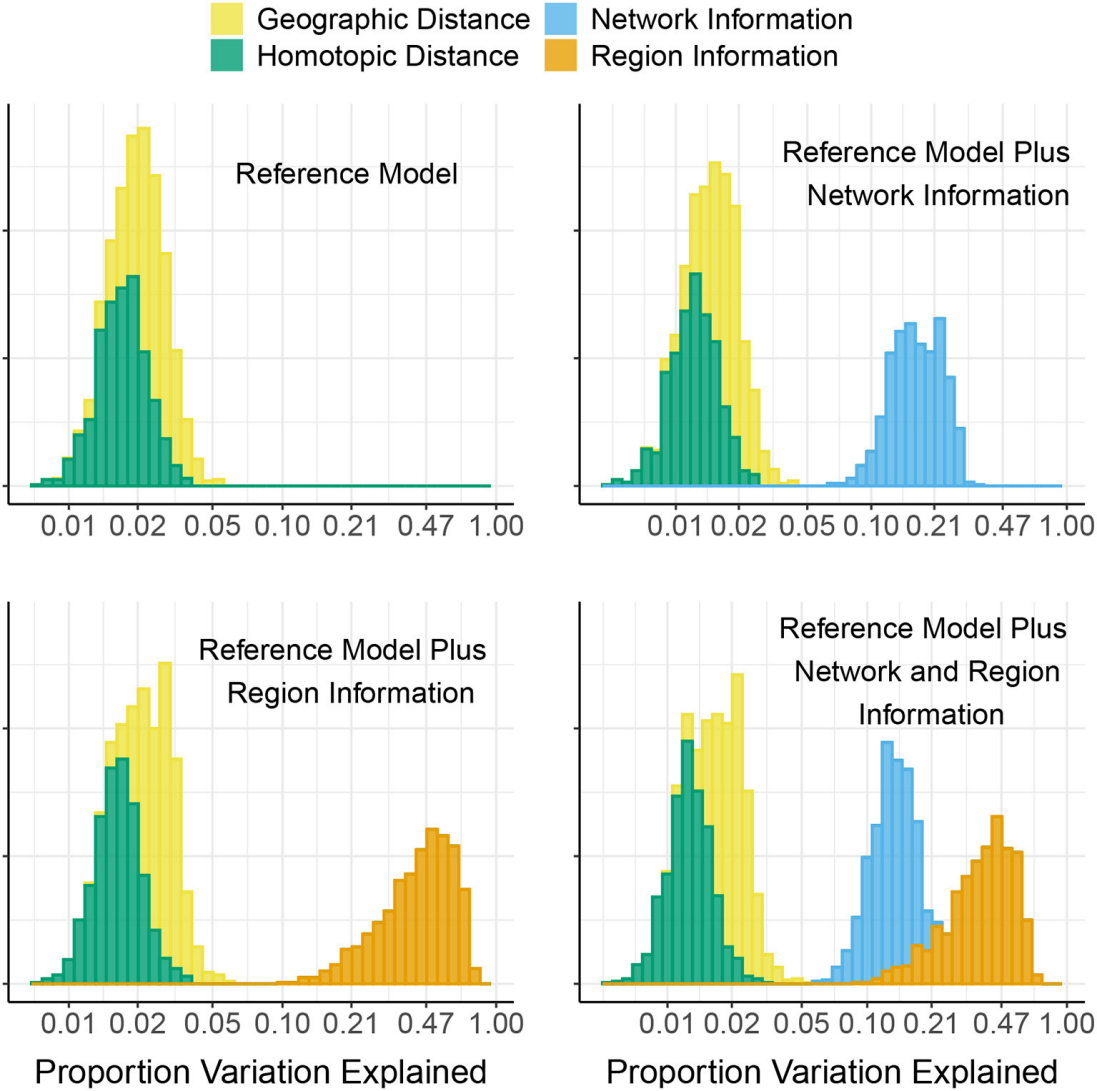
Partition of the proportion of variation in connectivity in the HCP data explained by our connectivity regression models. Each histogram shows the proportion of variation explained by each group of predictors used in the given model, for each of the 470 subjects and two sessions in the HCP data. Groups of predictors are: geographic distance between pairs of regions, homotopic distance between pairs of regions, network membership terms (indicating which functional network or pair of functional networks the regions belong to), and a region term for each ROI. Note that the *x*-axis is linear on the log_10_ scale.

**Figure 4:**
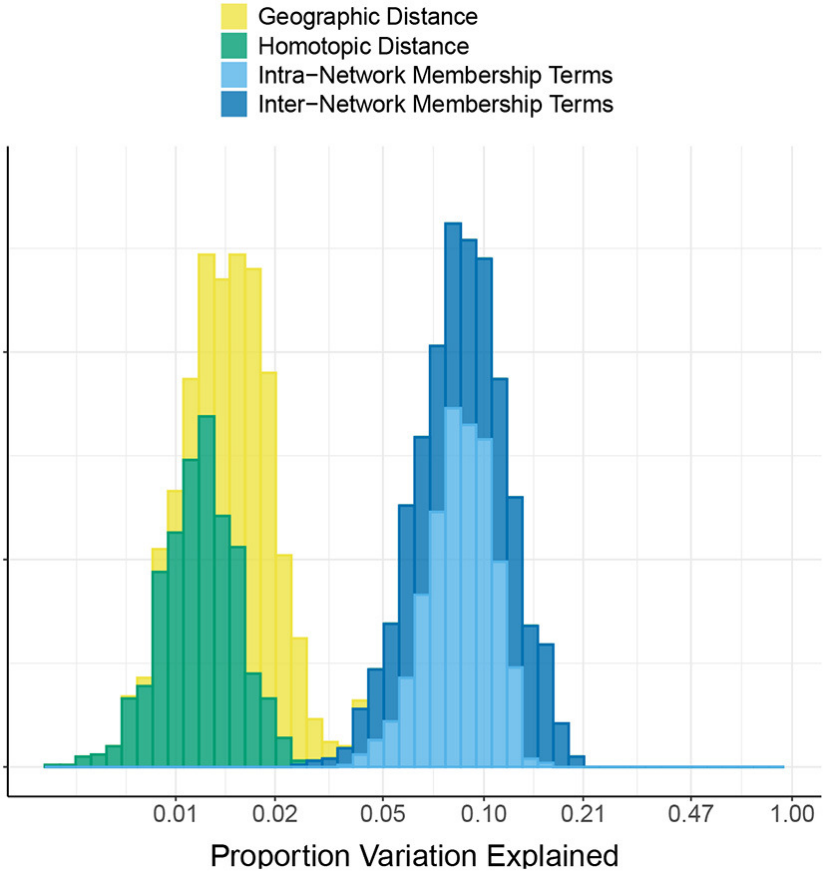
Comparison of intra- vs. inter- network terms in the HCP data. The histogram shows the proportion of variation in connectivity in the HCP data explained by each of the following groups of predictors: geographic distance between pairs of regions, homotopic distance between pairs of regions, intra-network terms (indicating that both regions are members of the same functional network), and inter-network terms (indicating that the two regions are members of a pair of different functional networks). Note that the *x*-axis is linear on the log_10_ scale.

Figure 2 shows the proportion of variation in connectivity explained in the main model (Equation 1), for each subject and session. This proportion varies between 0.22 and 0.85 across subjects and sessions, with a median of 0.56. Figure 3 shows the proportion of variation explained by each type of predictor (color) in each of our connectivity regression models (panel), for each subject and session. It is interesting to note that these distributions are somewhat insensitive to the model. For example, the distribution for network membership terms remains fairly similar whether region terms are included in the model or not. We also assessed, in the “Reference model + networks” model, whether intra-network or inter-network terms account for more variation explained. Figure 4 shows the distributions of proportion variation explained by geographic distance, homotopic distance, intra-network terms, and inter-network terms. Table 1 gives the means over subjects and sessions for the proportion of variation explained by each group of predictors in these models, as well as for the per-predictor relative importance of each group. As a group, the region terms had the largest contribution, explaining on average roughly 40% of the variability in the main model. However, in terms of per-predictor relative importance, region terms were the least important, while homotopic distance and geographic distance were most important by this metric. That is, geographic distance is a more important predictor (on average) than a single region term for one ROI; however, collectively the 268 region terms are more important than geographic distance. The proportion of variation explained by intra-network terms is similar to the proportion explained by inter-network terms. However, the per-predictor relative importance for the group of intra-network terms is three times as high; that is, on average, each intra-network term explains three times as much variation as a single inter-network term.

**Table 1:** Proportion of variation in connectivity in the HCP data explained by groups of predictors (P.V.E.), and per-predictor relative importance × 100 of each group (P.P.R.I.).

		**Group of predictors**				
**Model**		**Geography**	**Homotopy**	**Networks**	**Regions**	**Total**
Reference model	P.V.E.	0.025	0.018	–	–	0.043
	P.P.R.I.	1.227	1.835	–	–	
Reference model + networks	P.V.E.	0.018	0.013	0.177	–	0.208
	P.P.R.I.	0.916	1.278	0.492	–	
Reference model + regions	P.V.E.	0.027	0.017	–	0.451	0.496
	P.P.R.I.	1.364	1.745	–	0.169	
Reference model + networks + regions	P.V.E.	0.021	0.013	0.133	0.389	0.557
	P.P.R.I.	1.072	1.327	0.370	0.150	
		**Geography**	**Homotopy**	**Intra-**	**Inter-**	**Total**
				**network**	**network**	
Intra- vs. Inter-networks	P.V.E.	0.018	0.012	0.086	0.093	0.208
model	P.P.R.I.	0.886	1.236	1.070	0.343	

Values are averaged over 470 subjects and two sessions. One subject-session was not included in results for “Reference model + networks + regions” due to rank deficiency that occurred for that subject's data in one of the submodels used to compute proportion of variation explained.

Table 2 shows the point estimate of discriminability, $\hat{\delta }$, using our models and under the edge-at-a-time and edge distribution approaches. Our main connectivity regression model obtains a discriminability of 0.78, comparably as high as the edge-at-a-time approach (0.86). As expected, discriminability is lower using connectivity regression models that have a more limited set of covariates. The edge distribution approach obtains the same discriminability as our smallest connectivity regression model, with a value of 0.66. For a point of comparison, we can consider the values of discriminability that would be obtained if each subject's Day 1 measurement were randomly paired with the Day 2 measurement of a different subject rather than their own Day 2 measurement. Over different permutations of the Day 2 measurements in the HCP data, the average value of discriminability was 0.50, and 95% of values were in the interval (0.47, 0.52).

**Table 2:** Estimates of discriminability for the HCP data under three approaches.

**Approach**		**Discriminability**
Edge distribution		0.657
	Reference model	0.658
Partial	Reference model + networks	0.712
localization	Reference model + regions	0.773
	Reference model + networks + regions	0.781
Edge-at-a-Time		0.860

The edge-at-a-time approach uses the vector of Fisher Z-transformed correlations ***Z*** as the measurement. The partially localized approach uses the vector of fitted values $\hat{Z}$ from the connectivity regression model, while the edge distribution approach uses the (unordered) set *Ƶ* of correlations. For the ordered vectors, Euclidean distance is used; for the edge distribution approach, the 2-Wasserstein distance on the empirical distribution of correlations is used.

## 4. Discussion

In this paper, we propose the use of subject-level regression models to characterize functional connectivity while only using partial location information. These models use the Fisher-transformed correlation between a pair of regions (ROI's or voxels) as the outcome and characteristics of the region-pair as covariates. Our partially localized approach offers an alternative to edge-at-a-time approaches (which do not summarize the data and typically involve a large number multiple comparisons) on the one hand, and the edge distribution approach (which arguably over-summarizes the connectivity data, disregarding all location information) on the other.

We highlight two potential uses for these models. First, on the subject level, we can study the relative importance of different covariates toward explaining variability in each subject's connections. Here we have investigated this in the HCP data. Second, our models provide a way of summarizing subject-level connectivity through the fitted regression coefficients. Our approach could then be extended to compare connectivity between different groups of subjects, for example through a mixed effects model. Other functional connectivity approaches summarize subject-level connectivity via decomposition (for example, using independent components analysis, or ICA (Calhoun et al., 2009; Erhardt et al., 2011; Calhoun and Adali, 2012; Risk et al., 2014; Mejia et al., 2023) or using a collection of graph-theoretic metrics (Brier et al., 2014; Tao et al., 2020) before comparing across subjects. Our approach provides an additional way to summarize connectivity via interpretable parameters. Investigating the performance of our models for this application will be a direction for future research. A potential limitation is the loss of information incurred with our partial localization approach. Therefore, a question will be whether the information retained captures important differences between groups of subjects in specific applications, such as between patients in different arms of a given study.

As a way of exploring the amount of information captured by our approach in the context of the HCP data, we studied data repeatability. Our connectivity regression approach has comparably as high discriminability as the edge-at-a-time approach that uses full location information. However, this result should be taken with a grain of salt, since data repeatability is a statement about measurement variance and distinctness of a trait, regardless of the measure's utility as a biomarker. For example, images of human fingerprints are highly repeatable but contain no meaningful biological information. In addition, discriminability is not insensitive to demographics that impact measurements. For example, a dataset with a mixture of older and younger subjects will have higher discriminability than one with only subjects from a single age group (Wang et al., 2021). Thus, having high repeatability does not directly indicate that a measurement could be used as a biomarker. However, high repeatability is at least a desirable property; and the fact that our approach results in high discriminability even though the subjects compared here are all healthy controls is a promising indication of how much information can be captured through connectivity regression models.

**Figure 5:**
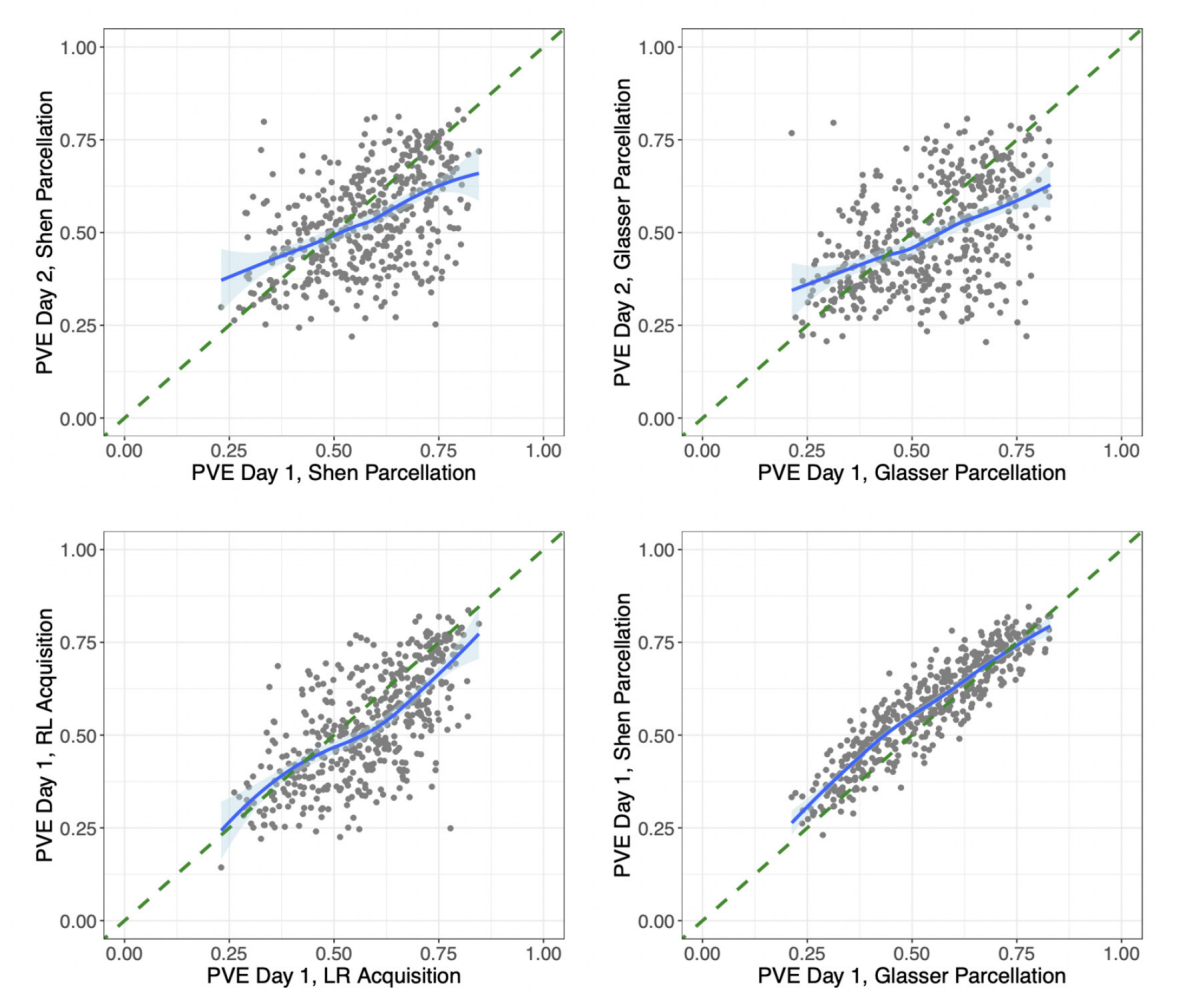
Differences in proportion variation explained by session, acquisition method, and parcellation/model. **(Upper left)** For each subject in the HCP data, we compare the proportion of variation explained by the same subject-level connectivity regression models fit on their data from Day 1 vs. on Day 2, both with left-to-right phase encoding and using the Shen parcellation. **(Upper right)** Proportion of variation explained by the same models fit on each subject's data from Day 1 vs. on Day 2, both with left-to-right phase encoding and using the Glasser parcellation. **(Lower left)** The proportion of variation explained by the same models (both using the Shen parcellation) fit on each subject's data from two different sessions on Day 1, one with left-to-right (LR) and the other with right-to-left (RL) phase encoding. **(Lower right)** The proportion of variation explained by two different models fit on data from the same session (Day 1 with LR phase encoding), extracted using the Glasser parcellation vs. the Shen parcellation. The model fit on the Shen-parcellated data uses geographic distance, homotopic distance, region terms for 268 ROI's, and network membership terms for eight networks. The model fit on the Glasser-parcellated data uses a homotopy indicator term, region terms for 360 ROI's, and network membership terms for 22 networks. The correlations based on data from different days are 0.50 and 0.48. The correlation from data from different scans on the same day is 0.67, and the correlation from different parcellation/models for data from the same scan is 0.91.

We also investigated the proportion of variation explained (PVE) by our models. In our main model, this ranged across subjects and sessions from extremely high (85% of the variation in connectivity) to quite low (22% of variation in connectivity). We found that subjects with a lower PVE for a given scan also tended to have weaker connectivity and lower variability in their connection strengths from that scan. However, the low PVE for some subjects did not seem to be due to quality control issues with these subjects' scans. To look for signs of whether the low PVE for some subjects could be due to poor functional alignment, we also compared each subject's PVE across different scans. Figure 5 compares the PVE in models fit on data from Day 1 vs. Day 2 for each subject in the HCP data. While some subjects have a low PVE for both days, there are also many subjects who have a low PVE on one day but a considerably higher PVE on the other day. This could indicate that low PVE for certain sessions/subjects may be due in large part to session- and subject-specific noise rather than biological variation across subjects in their functional alignment (though the comparison of sessions does not take into account the possibility of any intra-subject variation in functional alignment across sessions). Figure 5 also compares the PVE in models fit on data from two different scans on the same day for each subject (with left-to-right vs. right-to-left phase encoding), as well as the PVE from two different models fit on data from the same scan but extracted with two different parcellations. It is interesting to note the high discrepancy within subjects across days, where subjects can differ by up to 0.5 between technical replicates. In contrast, discrepancy was much lower within subjects over acquisition types and over atlas within the same day.

In our analysis, it is not surprising that region terms and network membership terms explained a large proportion of variability in connectivity. Region-level connectivity has significance related to both biology and nuisance reduction. Biologically, it is hypothesized that specific foci are “hubs” of network activity (see Van Den Heuvel and Sporns, 2011, for example). A benefit of the proposed approach is that it does not require hub locations to be consistent across subjects. From a nuisance perspective, any contaminant that increases or decreases connectivity in a spatially varying manner would receive some benefit from region adjustment in such a regression model. Similarly, the intercept includes both nuisance and possibly real effects, conflating global nuisance effects and increased overall connectivity, and is related to global signal regression (Liu et al., 2017). Network membership terms are unsurprising as having strong effects on connectivity since they are somewhat circularly used in this application. That is, networks are exactly sets of ROI's that exhibit strong inter-region correlations consistently across subjects (in the sample used by Finn et al., 2015).

In this paper, we illustrate our approach using data that had been registered to a common template-space. In the Supplementary material, we discuss how our approach could also be applied with data that is left in subject-space, in settings where one or several labeled templates are warped into subject space. We also explore how warping a template into subject-space, vs. registering subjects' data into standardized template-space, could impact our connectivity regression models. Future research will investigate extensions of our approach to subject-space data without labeled templates, including voxel-level data. In developing the extension of our approach to voxel-level analyses, the primary issue will be computational, since the number of correlations grows at the rate of the number of voxels squared, leading to hundreds of millions of correlations needing to be modeled.

Finally, we note that the analysis approach used here is not entirely novel. Intra-subject graph estimation combined with inter-subject analyses of graph metrics is a common method of analysis (see Wang et al., 2010), including the evaluation of repeatability (Braun et al., 2012; Andellini et al., 2015). However, weighted graph analyses in a regression model using these terms, along with measuring proportional variation explained, are less explored. We further believe that extending these methods to whole-brain analyses is possible and would lead to novel subject-level summaries of connectivity. In addition, these analyses were admittedly exploratory and approached without a priori hypotheses. An interesting future direction would be to further investigate model validation on repeatedly measured subjects and contrast model summaries across phenotypes such as disease status.

## Data availability statement

Publicly available datasets were analyzed in this study. This data can be found at: http://www.humanconnectome.org.

## Ethics statement

Ethical approval was not required for the study involving humans in accordance with the local legislation and institutional requirements. Written informed consent to participate in this study was not required from the participants or the participants' legal guardians/next of kin in accordance with the national legislation and the institutional requirements.

## Author contributions

BC conceived the ideas for this work. BS carried out the analysis with the help and supervision of BC. BS, BC, and ML wrote the manuscript with the help of YZ. All authors contributed to the article and approved the submitted version.
