# Supplementary material for: Regression models for partially localized fMRI connectivity analyses
Source: Front Neuroimaging. 2023 Nov 13;2:1178359. doi: 10.3389/fnimg.2023.1178359 (PMC10679340; doi:10.3389/fnimg.2023.1178359)
Supplement: Supplementary file 1 [file Presentation_1.pdf]

Supplementary Material

APPENDIX

Additional analyses

In order to investigate how the use of different parcellations may impact results when using connectivity regression models, we also analyze data from the same Human Connectome Project (HCP, Van Essen et al., 2013) subjects and sessions, but extracted using the Glasser parcellation with 360 ROI's organized into 22 networks (Glasser et al., 2016). In this analysis we use two connectivity regression models: model 'GL-HIIN' has an intercept, an indicator for whether the pair of regions are homotopic, 22 intra-network membership terms indicating that the two regions are in the same network, and 230 inter-network membership terms indicating that the regions are in different networks. Model 'GL-HNR' has an intercept, homotopy indicator, 252 (intra- and inter-) network membership terms, and 352 region terms (after removing terms to obtain a full-rank design matrix). Figure S1 shows the proportion of variation explained by each group of predictors in each of these models, for each subject and session. Table S1 shows the mean across subjects and sessions of the proportion of variance explained and the per-predictor relative importance of groups of predictors in these two models. The group of network membership terms and the group of region terms each explained approximately one quarter of the variation. The homotopy indicator has the highest per-predictor relative importance by far in both models. As with the Shen parcellation, intra-network terms have a higher per-predictor relative importance than inter-network terms. Table S2 shows discriminability using these models versus the edge-at-a-time and edge distribution approaches. The level of discriminability is very similar to that seen for data using the Shen parcellation.

To further assess sensitivity to the choice of parcellation, we also constructed a parcellation where ROI's were obtained as the union of two ROI's from the Shen parcellation within the same network and in the same hemisphere (or as a single ROI from the Shen parcellation, for regions that could not be paired). Regional time courses for the resulting 139 ROI's were extracted, and our main model (equation (1) in the main text) was fit on the resulting regional data. Results are shown in Tables S1 and S2, and are fairly similar to results seen using the Shen parcellation, except that network membership terms explain a higher proportion of variation under this coarsened parcellation than with the Shen parcellation.

As a negative control, we fit an alternate version of our main model where we assigned random network labels, rather than using the functional networks of Finn et al. (2015). As expected, under this model, network membership terms explained a very small proportion of variation: 0.010, compared to 0.133 in our main analysis.

Our main analysis was fit on data acquired with left-to-right phase encoding. We additionally fit our main model on the same subjects' right-to-left phase encoding data. The mean proportion of variation explained was 0.550, partitioned as: geography 0.022, homotopy 0.013, network membership terms 0.133, and region terms 0.382. This was almost identical to the partition from the left-to-right phase encoding data.

Impact of transforming labeled templates into subject-space

Here we consider how warping one or more templates into subject-space, versus registering subjects' data into a common template-space, could impact our proposed connectivity regression analysis approach. We focus on the setting where a labeled template is transformed into subject-space and where, additionally,

the ROI's in the labeled template have been partitioned into functional networks in advance using data from an independent group of subjects. (That is, the assignment of ROI's to functional networks is not subject-specific.) Consider a given transformation from a labeled template into subject-space. Since they do not depend on the coordinates for each region, the region terms and the network membership terms in our model are not impacted by this transformation. On the other hand, predictors such as geographic distance and homotopic distance generally would be impacted (though not under certain transformations such as rotations, reflections, or translations). Given a fixed labeled template, regional time courses are not impacted by a coordinate transformation. However, using multi-atlas registration, the choice of template will impact regional time courses and the connectivity outcome data in our models.

Next we use a simulation approach to explore the impact of transformations, such as those that may be used to map a labeled template to subject-space. We chose two examples of non-linear transformations, and we constructed simulated subject-space predictors by applying these transformations to the region-centers from the Shen parcellation. We then compare the results from fitting our main connectivity regression model on the HCP data with the original predictors (in common template-space) versus the simulated subject-space predictors. The transformations that we used were chosen for interpretability, with a goal of learning about mechanisms by which transformation into subject-space can impact results. Specifically, ϕ_1 is a transformation that enlarges the right hemisphere but not the left, and ϕ_2 enlarges the front half of the brain but does not affect the rear half. In particular, homotopic distance between the region-centers should be less impactful under the simulated predictors having less left-right symmetry (that is, the simulated predictors obtained using the ϕ_1 transformation). The analysis presented here gives a first illustration of some of the mechanisms by which transformations between common template-space and subject-space can impact connectivity regression models; a more comprehensive investigation of this issue in future research could study a wider range of transformations as well as taking into account multi-atlas registration.

Figure S2 shows the region-centers in the Shen parcellation (in common template-space), as well as the simulated subject-space region-centers obtained by applying transformations ϕ_1 and ϕ_2. Figure S3 shows term plots for an example subject from the HCP data for the following model:

$$\begin{aligned} Z(j,j') = \beta_0 + s_0(\texttt{GeogDist}(j,j'); \texttt{Hem}_0) + s_1(\texttt{GeogDist}(j,j'); \texttt{Hem}_1) \\ + s_2(\texttt{HomotopDist}(j,j'); \texttt{Hem}_1) + \epsilon(j,j') \end{aligned} \tag{S1}$$

As we would expect, homotopy has a dampened impact in the simulated subject-space obtained using ϕ_1. Table S3 shows the proportion of variation explained in our main model using the simulated subject-space predictors. Notably, the relative importance of homotopy is diminished in the simulated subject-space using ϕ_1, with relative importance only two thirds as high as in our main analysis.

REFERENCES

Finn, E. S., Shen, X., Scheinost, D., Rosenberg, M. D., Huang, J., Chun, M. M., et al. (2015). Functional connectome fingerprinting: identifying individuals using patterns of brain connectivity. *Nature Neuroscience* 18, 1664–1671

Glasser, M. F., Coalson, T. S., Robinson, E. C., Hacker, C. D., Harwell, J., Yacoub, E., et al. (2016). A multi-modal parcellation of human cerebral cortex. *Nature* 536, 171–178. doi:https://doi.org/10.1038/nature18933

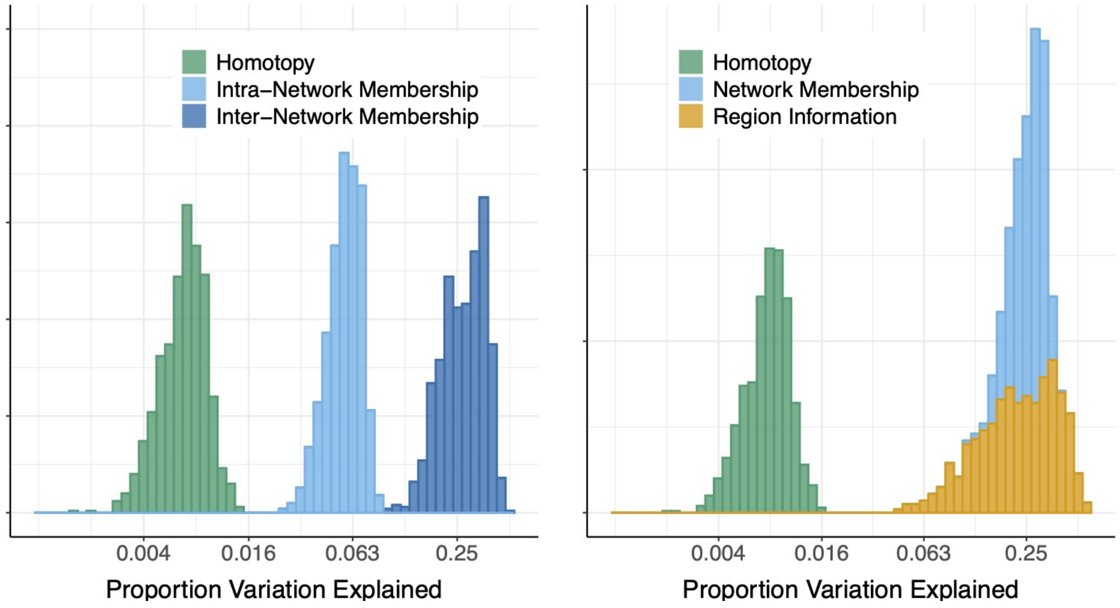

Figure S1. Proportion of variation explained using the Glasser parcellation. The histograms show the proportion of variation in connectivity in the HCP data explained by each group of predictors in models using the Glasser parcellation. Left panel: the first model uses a homotopy indicator, a group of 22 intra-network terms, and a group of 232 inter-network terms. Right panel: the second model uses a homotopy indicator, a group of 252 network membership terms, and a group of 352 region terms. Note that the x-axis is linear on the $\log_{10}$ scale.

Table S1. Proportion of variation in connectivity explained (P.V.E.) by groups of predictors, and per-predictor relative importance $\times 100$ (P.P.R.I.) of each group, in models fit using alternate parcellations. Model '139-ROI' is fit on HCP data extracted using a parcellation with 139 ROI's, which was obtained by coarsening the Shen parcellation. Models 'GL-HIIN' and 'GL-HNR' are fit on HCP data from the same subjects and sessions, extracted using the Glasser parcellation. Values are averaged over subjects and sessions.

Model		Group of predictors				
		Geography	Homotopy	Networks	Regions	Total
139-ROI	P.V.E.	0.029	0.020	0.194	0.394	0.638
	P.P.R.I.	1.446	2.046	0.540	0.152	
			Homotopy	Intra-Network	Inter-Network	Total
GL-HIIN	P.V.E.		0.007	0.057	0.280	0.345
	P.P.R.I.		0.700	0.261	0.122	
			Homotopy	Networks	Regions	Total
GL-HNR	P.V.E.		0.008	0.259	0.249	0.516
	P.P.R.I.		0.798	0.103	0.071	

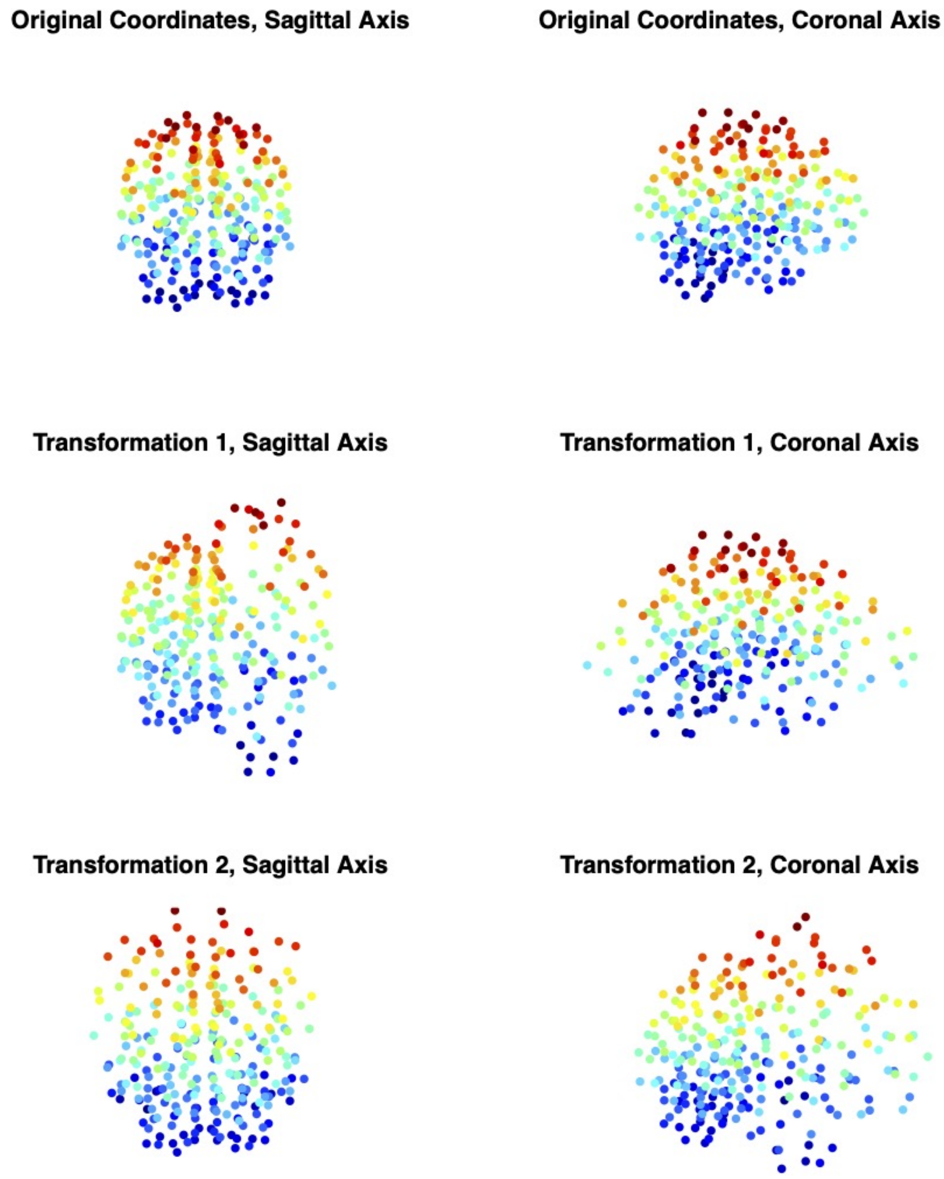

Figure S2. Visualization of simulated subject-space region-centers. The top row shows the centers of the 268 regions in the Shen parcellation in common template-space. The middle and lower rows show region-centers for simulated subject-space data, where the simulated region-centers were obtained by applying transformations to the Shen region-centers. The two transformations enlarge the right hemisphere while leaving the left hemisphere fixed (middle row), or enlarge the front half of the brain only (lower row).

Van Essen, D. C., Smith, S. M., Barch, D. M., Behrens, T. E., Yacoub, E., Ugurbil, K., et al. (2013). The WU-Minn human connectome project: an overview. *Neuroimage* 80, 62–79

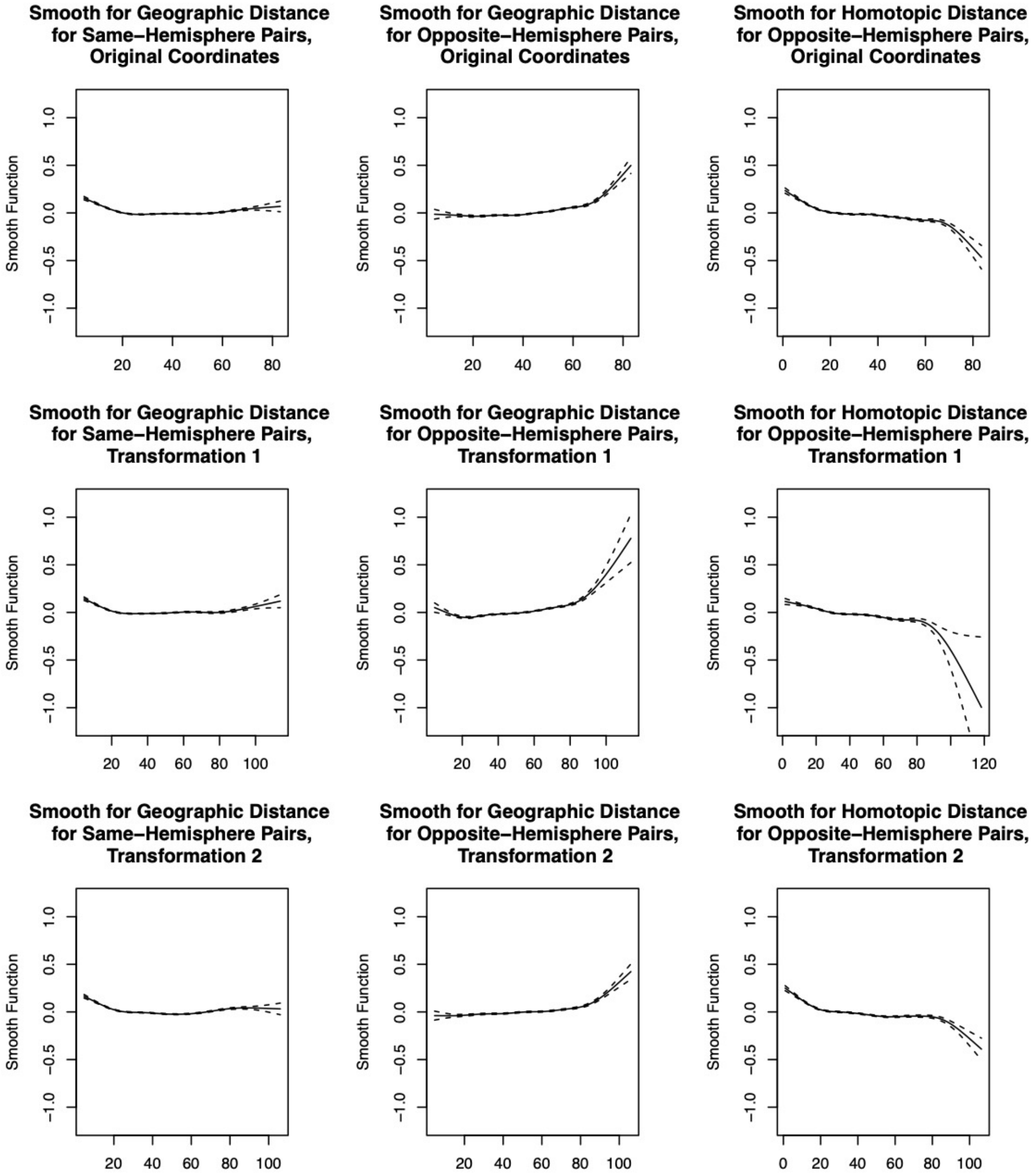

Figure S3. Term plots of a model fit in simulated subject-space. The upper row shows term plots for geographic distance and homotopic distance from fitting an example subject's data in common template-space. The middle and lower rows show term plots from fitting the same subject's data, but with predictors based on simulated subject-space region-centers. In the original coordinates, region-pairs who were symmetrically opposite (with homotopic distance close to zero) had stronger connectivity, after adjusting for geographic distance. However, as expected, this effect is diminished in a simulated subject-space with a lack of symmetry between the left and right hemispheres (middle row). The effect is not altered in a simulated subject-space where the front half of the brain is larger but the left and right hemispheres remain symmetric (bottom row).

Table S2. Estimates of discriminability for the HCP data with alternate parcellations. The model 'GL-HIIN' uses a homotopy indicator, intra-network terms, and inter-network terms. The model 'GL-HNR' uses a homotopy indicator, network membership terms, and region terms. The 'ROI-139' parcellation was obtained by coarsening the Shen parcellation; the model fit using this parcellation has geographic distance, homotopic distance, region terms, and network membership terms.

Parcellation	Approach	Model	Discriminability
Glasser	Edge Distribution		0.651
	Partial Localization	GL-HIIN	0.713
		GL-HNR	0.754
	Edge-at-a-Time		0.836
139-ROI	Edge Distribution		0.657
	Partial Localization		0.765
	Edge-at-a-Time		0.823

Table S3. Proportion of variation explained (P.V.E.) by simulated subject-space predictors, and per-predictor relative importance $\times 100$ for each group of predictors (P.P.R.I.). The first model uses simulated subject-space region-centers that were obtained from the Shen region-centers via a transformation (ϕ_1) that enlarges the right hemisphere but not the left, such that the simulated subject-space coordinates have disrupted left-right symmetry. The second model uses simulated region-centers obtained using a transformation (ϕ_2) that enlarges the front half of the brain but not the back, impacting geographic relationships among the region-centers but preserving left-right symmetry. Also shown is the ratio of per-predictor relative importance in these models versus in the main analysis using the Shen region-centers (Ratio vs Shen). Values are averaged over 470 subjects and two sessions. Note: two subject-sessions were excluded from the analysis for the ϕ_1 model, and one subject-session was excluded from the analysis for the ϕ_2 model due to a non-full-rank design matrix in one of the submodels used for the proportion variation explained computation.

		Group of predictors				
Model		Geography	Homotopy	Networks	Regions	Total
ϕ_1-transformed region-centers	P.V.E.	0.020	0.009	0.135	0.391	0.554
	P.P.R.I.	0.989	0.931	0.375	0.150	
	Ratio vs Shen	0.898	0.690	1.013	1.023	
ϕ_2-transformed region-centers	P.V.E.	0.021	0.014	0.132	0.388	0.554
	P.P.R.I.	1.028	1.352	0.367	0.149	
	Ratio vs Shen	0.932	1.002	0.996	1.015	
